# Grey and white matter atrophy 1 year after stroke aphasia

**DOI:** 10.1093/braincomms/fcac061

**Published:** 2022-03-17

**Authors:** Natalia Egorova-Brumley, Mohamed Salah Khlif, Emilio Werden, Laura J. Bird, Amy Brodtmann

**Affiliations:** 1 The Florey Institute of Neuroscience and Mental Health, Melbourne, Australia; 2 The University of Melbourne, Melbourne, Australia

**Keywords:** atrophy, corpus collosum, inferior frontal gyrus, post-stroke aphasia, superior longitudinal fasciculus

## Abstract

Dynamic whole-brain changes occur following stroke, and not just in association with recovery. We tested the hypothesis that the presence of a specific behavioural deficit after stroke would be associated with structural decline (atrophy) in the brain regions supporting the affected function, by examining language deficits post-stroke. We quantified whole-brain structural volume changes longitudinally (3–12 months) in stroke participants with (*N* = 32) and without aphasia (*N* = 59) as assessed by the Token Test at 3 months post-stroke, compared with a healthy control group (*N* = 29). While no significant difference in language decline rates (change in Token Test scores from 3 to 12 months) was observed between groups and some participants in the aphasic group improved their scores, stroke participants with aphasia symptoms at 3 months showed significant atrophy (>2%, *P* = 0.0001) of the left inferior frontal gyrus not observed in either healthy control or non-aphasic groups over the 3–12 months period. We found significant group differences in the inferior frontal gyrus volume, accounting for age, sex, stroke severity at baseline, education and total intracranial volume (Bonferroni-corrected *P* = 0.0003). In a subset of participants (aphasic *N* = 14, non-aphasic *N* = 36, and healthy control *N* = 25) with available diffusion-weighted imaging data, we found significant atrophy in the corpus callosum and the left superior longitudinal fasciculus in the aphasic compared with the healthy control group. Language deficits at 3 months post-stroke are associated with accelerated structural decline specific to the left inferior frontal gyrus, highlighting that known functional brain reorganization underlying behavioural improvement may occur in parallel with atrophy of brain regions supporting the language function.

## Teaser

Egorova-Brumley *et al*. report that aphasia at 3 months post-stroke is associated with increased brain volume loss in the left inferior frontal gyrus compared with non-aphasic and healthy controls, highlighting that brain reorganization involves not only changes associated with functional improvement but also atrophy in the regions supporting impaired functions.

## Graphical Abstract

**Graphical Abstract:**
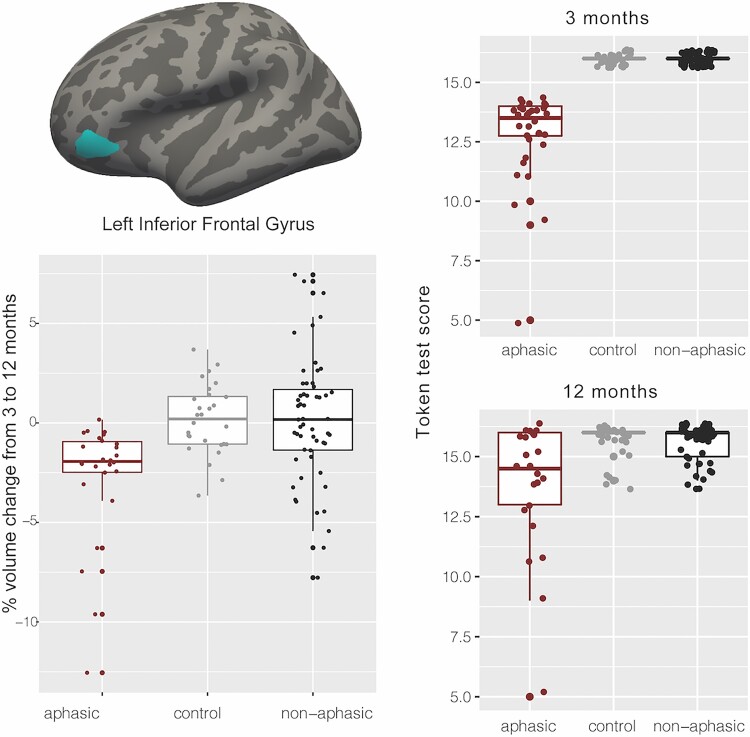

## Introduction

Network-wide changes after stroke have been well-described, including neuroinflammation, microglial activation and eventual Wallerian degeneration.^1^ The concept of post-stroke neurodegeneration as a neural network disorder is increasingly described: the acute infarct triggering a cascade of events leading to brain atrophy and neurodegeneration.^2,3^ In other neurodegenerative disorders, brain atrophy signatures have been identified along the specific functional and structural networks.^4–6^ For example, in amnestic mild cognitive impairment—often conceptualized as prodromal Alzheimer’s disease—memory and attention deficits were associated with distinctive brain atrophy patterns that differed from the more global brain atrophy as seen in healthy brain ageing.^7^

In progressive neurodegenerative diseases, behavioural deficits typically emerge gradually over time, and are often preceded by the underlying brain atrophy.^8^ Both cognitive decline and atrophy continue over time. In stroke, however, specific behavioural deficits tend to emerge at the time of, or soon after, stroke. They are associated with lesion-related disruption of specific functional and structural connections, rather than accumulated brain atrophy. This provides an opportunity to examine the extent of atrophy, which is potentially caused by the disruption of a particular function early after stroke.

Aphasia affects 21–38% of acute stroke patients,^9^ with the greatest recovery taking place in the first 3 months post-stroke.^10^ After that time, improvement is less marked and plateaus around 1 year.^10^ Most researchers using brain imaging in aphasia have focused on understanding brain reorganization associated with recovery after stroke.^11–13^ However, post-stroke aphasia offers a unique opportunity to test the hypothesis of brain atrophy along functional circuits. We predicted that in stroke patients who presented with aphasia at 3 months (likely caused by the disruption of the brain regions supporting the language function), we would observe brain atrophy in the regions associated with language in the first year after stroke and that it would not be present in non-aphasic stroke patients or healthy controls.

## Materials and methods

### Participants

Participants with ischaemic stroke (first-ever or recurrent) were recruited from the Stroke Units at three Melbourne hospitals: Austin Hospital, Box Hill Hospital and the Royal Melbourne Hospital as a part of the Cognition And Neocortical Volume After Stroke (CANVAS) study.^14^ Age- and sex-matched healthy control participants were recruited from the general population. Each hospital’s human research ethics committee approved the study, and participants provided consent in accordance with the Declaration of Helsinki.

From the total of 175 participants in the CANVAS study, 120 (91 stroke and 29 healthy control) were included in the analysis, based on the availability of the Token Test scores at 3 months and the 3- and 12-month imaging data, 55 were excluded as described below. Stroke participants scoring 14 and below on the Token Test (*N* = 32) were assigned to the aphasic stroke group. Stroke participants with a perfect score of 16, demonstrating no language comprehension deficit (*N* = 59), were assigned to the non-aphasic stroke group. Healthy control participants scoring 16 (*N* = 29) were allocated to the healthy control group. Stroke participants scoring 15 (above the threshold for aphasia diagnosis but below the perfect 16 score) (*N* = 27) and healthy control participants with any score below 16 (*N* = 11), as well as participants missing structural imaging data at 3 or 12 months (*N* = 17) were excluded.

### Outcome measures

The severity of participants’ strokes was assessed with the National Institutes of Health Stroke Scale (NIHSS), performed during hospital admission. Language ability was assessed using the 16-item short form of the Token Test, which screens for receptive disorders in aphasia.^15^ The cut-off score of 14 has been shown to be useful for identifying aphasic patients with 84% accuracy, with no distinction regarding the type of aphasia.^15^

Other language assessments included the Controlled Oral Word Association Test (COWAT)^16^, which measures animal category fluency and lexical fluency (letters F, A and S). In both fluency tests, participants were instructed to produce as many exemplars as possible within 1 min. Boston Naming Test^17^ was used to assess confrontational word retrieval.

In addition, we collected measures of verbal memory, working memory and attention with Hopkins Verbal Learning Test (HVLT)-delay task^18^ and Cogstate one-back and identification tasks (Cogstate Ltd., Melbourne, Australia), respectively, to control for non-language-specific cognitive impairments. All tests were conducted in English, and all participants were proficient in English; proficiency in other languages was not assessed.

### Imaging data acquisition

All images were acquired on a Siemens 3 T Tim Trio scanner (Erlangen, Germany) with a 12-channel head coil. As part of the ongoing longitudinal CANVAS study, participants were assessed at 3- and 12-months post-stroke. A high-resolution anatomical magnetization-prepared rapid acquisition with gradient echo (MPRAGE) scan was collected [volume of 160 sagittal slices with 1 mm isotropic voxels, time of repetition (TR) = 1900 ms, echo time (TE) = 2.55 ms, 9° flip angle, 100% field of view in the phase direction and 256 × 256 acquisition matrix]. A high-resolution 3D Fluid Attenuated Inversion Recovery (SPACE-FLAIR) image was acquired (with 160 1 mm thick sagittal slices, TR = 6000, TE = 380 ms, 120° flip angle, 100% field of view in the phase direction and 256 × 254 acquisition matrix) to delineate lesions. Sixty diffusion-weighted images (*b* = 3000 s/mm²), and eight volumes without diffusion weighting (*b* = 0), were obtained with 2.5 mm × 2.5 mm × 2.5 mm isotropic voxels.

### Lesion analysis

**Figure 1:**
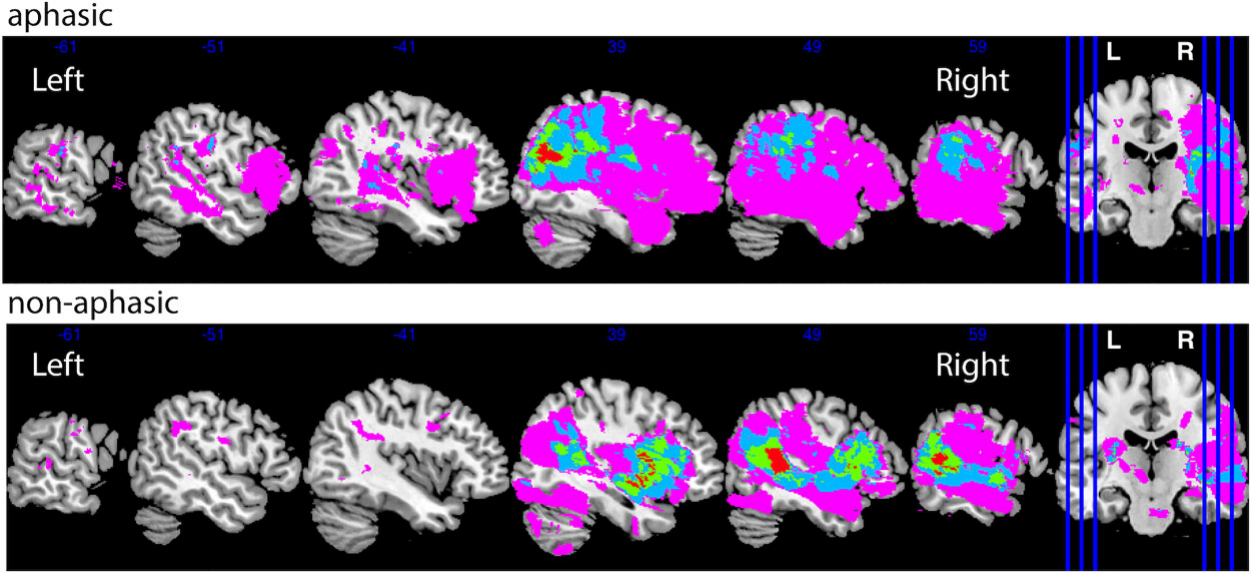
**Lesion distribution by group.** The number of participants with overlapping lesions ranges between 1 and 4.

Lesions were manually traced on the high-resolution FLAIR image. A stroke neurologist (A.B.) visually inspected and verified the manually traced images. Binary lesion masks were created and normalized to the MNI template using the Clinical Toolbox SPM extension.^19^ Lesion overlap images were prepared using the MRIcron software.^20^ We created the lesion overlap maps for stroke participants with and without aphasic symptoms (Fig. 1).

### White matter hyperintensity analysis

Automated segmentation of white matter (WM) hyperintensities (WMHs) was performed using the Wisconsin WMH Segmentation (W2MHS) toolbox using FLAIR and T_1_ images as inputs.^21^ Manual corrections were performed to remove false positive voxels in the choroid plexus, brainstem and cerebellum when necessary. WMH load was calculated as percentage of total brain volume and reported in Table 1.

**Table 1:** Demographic variables by group

Variable	Aphasic stroke	Non-aphasic stroke	Healthy control	Aphasic versus healthy control, *P*-value	Non-aphasic versus healthy control, *P*-value	Aphasic versus non-aphasic, *P*-value	Test (two-tailed)
*N*	32	59	29				
Token Test at 3 months (mean, SD)	13 (2)	16 (0)	16 (0)				
Sex (*N* women, %)	10 (31%)	23 (39%)	10 (34%)	0.79	0.68	0.46	*χ* ^2^
*N* left-handed	1	3	3	0.26	0.36	0.66	*χ* ^2^
NIHSS baseline (median, range)	3 (0–15)	2 (0–7)	n/a	n/a	n/a	0.02	Mann–Whitney Wilcoxon test
Years of education (mean, SD)	11.37 (3.58)	13.1 (3.38)	14.62 (3.77)	<0.001	0.03	0.07	*t*-test
Age at 3 months (mean, SD)	72.46 (8.78)	65.57 (13.06)	69.51 (5.39)	0.12	0.05	<0.001	*t*-test
Total intracranial volume (TIV) at 3 months, ml (mean, SD)	1505 (170)	1478 (172)	1533 (126)	0.47	0.09	0.47	*t*-test
Lesion volume at 3 months, ml (mean, SD)	18 (48)	7 (14)	n/a	n/a	n/a	0.21	*t*-test
White matter hyperintensity (WMH) load at 3 months, %TIV (median, range)	0.34 (0.05–5.01)	0.43 (0.01–9.36)	0.42 (0.06–1.56)	0.48	0.18	0.76	*t*-test^a^

^a^ Statistical analysis performed on Log(WMH/TIV) values; WMH data available for 26 aphasic, 46 non-aphasic and 25 control participants.

### Statistical analyses

#### Grey matter volume statistical analysis

We automatically estimated structural volumes using the longitudinal stream^22^ in FreeSurfer v.6.0 (http://surfer.nmr.mgh.harvard.edu/). With this method, an unbiased within-subject template space and image^23^ are created using robust, inverse consistent registration. Subsequent pre-processing steps are based on the common information from the within-subject template and include skull stripping, Talairach transforms, atlas registration, creating spherical surface maps and parcellations.^22^ The unbiased template was created for each participant using the T_1_ MPRAGE scans collected at each time point. Tissue segmentations for individual participants were visually inspected and corrected. Volume estimates were computed for each longitudinal scan in all regions of the FreeSurfer default cortical and subcortical parcellations based on the Desikan-Killiany Atlas.^24^ The specific regions of interest (ROIs) used for the analysis are listed in Supplementary Table 1. The selection of ROIs covered most of the brain, so our approach can be considered pseudo-whole brain.

Group differences were examined using an ANCOVA with three groups (aphasia, non-aphasia and healthy control) performed for each ROI in Supplementary Table 1, controlling for age, sex, total intracranial volume (TIV) and years of education. Significance was set at a Bonferroni-corrected level of *P* < 0.0006, since we conducted 82 tests. For the ROIs showing significant volume differences (3–12 months) between groups, we performed pairwise comparisons, which in the two-stroke groups also controlled for NIHSS scores at baseline.

Finally, we calculated per cent change within each group in each significant ROI and used a one-sample *t*-test (two-tailed) to determine if the change was significant.

#### WM fixel-based statistical analysis

For the analysis of WM structure, we applied a fixel-based approach as in Egorova *et al.*^25^ which is sensitive to fibre tract-specific differences at a ‘fixel’ (‘fibre population within a voxel’) level to assess axonal loss across all WM fixels in the brain. The outcome metrics of this analysis are fibre density and fibre bundle cross-section. Fibre density is a metric sensitive to the total intra-axonal volume of axons aligning with a specific fibre population in each voxel compartment. Fibre cross-section is sensitive to individual differences in macroscopic fibre bundle cross-sectional size.^26^

Pre-processing of diffusion-weighted images included denoizing, removing Gibbs ringing artefacts, eddy-current distortion and motion correction, bias field correction and spatial upsampling. Following these pre-processing steps, WM fibre orientation distributions (FODs) were computed with single-shell three-tissue constrained spherical deconvolution (SS3T-CSD), with group averaged response functions for WM, grey matter (GM) and CSF obtained from the data themselves,^27,28^ using the MRtrix3Tissue (https://3Tissue.github.io), a fork of MRtrix3.^29^ All pre-processing was performed in the same way for both healthy control and stroke patients. Note that the lesions in stroke participants were not explicitly masked out, but thanks to the SS3T-CSD method, they were automatically characterized as a mixture of WM-like, GM-like and CSF-like signal. The WM FODs accurately quantify the amount of ‘intact’ WM, while contributions of other (pathological) tissues, such as stroke lesions or WMHs, and free water are accommodated in other model compartments.^30^

Longitudinal pre-processed diffusion data were available only for a subset of the original cohort, with *N* = 14 in the aphasic, *N* = 36 in the non-aphasic and *N* = 25 in the healthy control group. We performed statistical comparisons of fibre density and fibre cross-section for all WM fixels between groups (aphasic versus non-aphasic and aphasic versus healthy control), controlling for age, education and intracranial volume, using connectivity-based fixel enhancement^31^ for the fibre density (FD) and fibre cross-section (FC) metrics separately, using the difference images (12 minus 3 months). Significant fixels (FWE-corrected *P* < 0.05, non-parametric permutation testing over 5000 permutations) were then visualized on the population template. Statistical analysis steps were performed using the MRtrix3.^29^

Automated TractSeg tool was used to delineate the tracts where significant results were found in the whole-brain analysis, namely the left superior longitudinal fasciculus and the corpus callosum tracts to plot FD and FC values by group (aphasic, non-aphasic healthy control) and by time point (3 and 12 months). We extracted tracts of interest [CC_3 and the superior longitudinal fasciculus (SLF) III tracts in TractSeg] using the default pipeline (https://github.com/MIC-DKFZ/TractSeg) limiting the number of streamlines to 10 000, which proved to be sufficient to delineate each tract well.

### Data availability

The data that support the findings of this study are available on reasonable request from the corresponding author. Requests for raw and analyzed data will be reviewed by the CANVAS investigators to determine whether the request is subject to any intellectual property or confidentiality obligations.

## Results

### Behavioural results 3 months post-stroke

The aphasic and non-aphasic stroke groups were not significantly different in sex, handedness or years of education. They were, however, different on baseline NIHSS scores (more severe stroke in the aphasic group), and age (older participants in the aphasic group; see Table 1). Both stroke groups were not different from controls on sex and handedness. Handedness was not considered as covariate in our analyses due to very low numbers of left-handers in the sample—1 left-handed participant in the aphasic group (0.03%) and three in the non-aphasic group (0.05%). Healthy controls were better educated than both stroke groups and younger than non-aphasic stroke participants (Table 1). There was a slightly higher number of participants with right-hemisphere stroke lesions in our sample *N* = 53 (∼58%), compared with left-hemisphere *N* = 36 (∼39%), and bilateral *N* = 2 (∼2%) stroke lesions, as the CANVAS study did not specifically focus on aphasia. We have confirmed that there were no differences between the aphasic and non-aphasic groups in the proportion of left versus right-lateralized lesions (*X*^2^ = 1.91, *P* = 0.167), see Fig. 1.

By design aphasic group differed significantly from non-aphasic and healthy control groups on Token Test at 3 months. On other available language tests at 3 months (Boston Naming Test, COWAT animal and lexical fluency), the stroke aphasic group performed significantly worse than the non-aphasic stroke and healthy control groups. The non-aphasic group performed worse on the lexical fluency test than healthy controls, but was comparable with healthy controls in the Boston Naming Test and the semantic fluency test (animals), see Table 2.

**Table 2:** Language variables by group

Variable	Aphasic stroke	Non-aphasic stroke	Healthy control	Aphasic versus healthy control	Non-aphasic versus healthy control	Aphasic versus non-aphasic	Test (two-tailed)
Boston Naming Test score at 3 months (mean, SD)	22.81 (5.50)	26.45 (3.97)	27.66 (2.53)	<0.001	0.09	<0.001	*t*-test
COWAT animals score at 3 months (mean, SD)	14.6 (5.23)	19.84 (7.12)	20.62 (5.94)	<0.001	0.59	<0.001	*t*-test
COWAT FAS score at 3 months (mean, SD)	26.1 (13.66)	35.58 (10.71)	42.66 (11.07)	<0.001	0.01	<0.001	*t*-test
Boston Naming Test score at 12 months (mean, SD)	24.80 (3.79)	27.22 (2.89)	27.59 (3.08)	0.01	0.60	0.01	*t*-test
COWAT animals score at 12 months (mean, SD)	15.50 (4.40)	19.55 (5.35)	20.76 (5.33)	<0.001	0.33	<0.001	*t*-test
COWAT FAS score at 12 months (mean, SD)	27.96 (9.07)	38.09 (11.08)	42.76 (11.44)	<0.001	0.08	<0.001	*t*-test

### Language recovery between 3 and 12 months post-stroke

**Figure 2:**
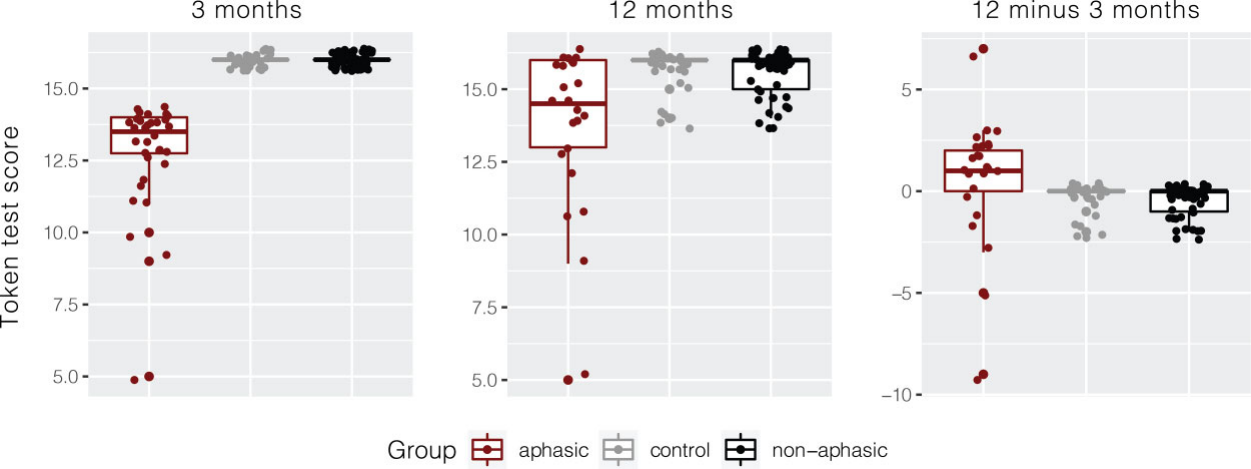
**Token Test results by group at 3 months, 12 months and the change between 12 and 3 months.** ANOVA on Token Test scores at 12 months *F*(2,105) = 4.25, *P* = 0.017, aphasic versus non-aphasic (*P* = 0.04), aphasic versus the healthy control (*P* = 0.02), non-aphasic versus healthy control (*P* = 0.99).

Token Test scores for 12 months were missing from eight participants in the aphasic group and six participants in the healthy control group, reflected in the total degrees of freedom for the reported analyses. Note that participants with missing Token Test data at 12 months were not excluded from the brain analyses. There were significant differences between the groups on the Token Test changes from 3 to 12 months, Fig. 2. The median increase in Token Test scores from 3 to 12 months in the aphasic group was +1 point (ranging from −9 to 7), however, given the threshold of 14 for the diagnosis of aphasia, for 50% of the participants (12 out of 24), this improvement meant that they were no longer aphasic.

Although the Token Test scores numerically improved between 3 and 12 months in the aphasic group (driven by a few participants), aphasic stroke participants continued to perform significantly worse than both non-aphasic stroke and healthy control participants at 12 months [*F*(2,105) = 4.25, *P* = 0.017, aphasic versus non-aphasic (*P* = 0.04), aphasic versus healthy control (*P* = 0.02), non-aphasic versus healthy control (*P* = 0.99)]. Furthermore, they continued to show worse performance compared with healthy controls and non-aphasic participants on the Boston Naming Test and the verbal fluency tests, Table 2.

### GM volume results 3–12 months post-stroke

**Figure 3:**
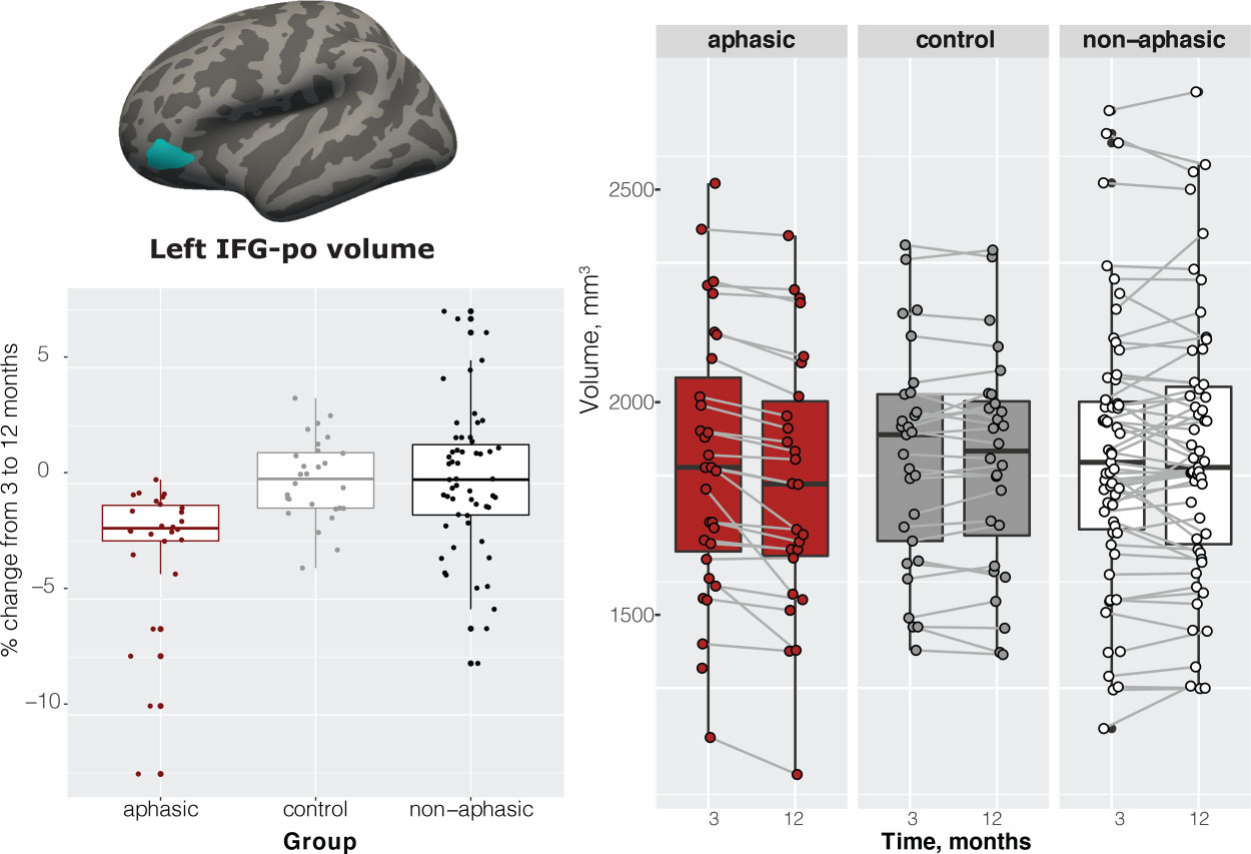
**GM imaging results.** Location of the left IFG, pars orbitalis (IFG-po); per cent change between 3 and 12 months by group; left IFG-po volume by time by group. ANOVA on 3–12 months volume change—*F*(2,108) = 8.84, *P* = 0.0003, aphasic versus healthy controls (*P* = 0.0033), aphasic versus non-aphasic stroke (*P* = 0.0003), healthy control versus non-aphasic (*P* = 0.9866).

Of all the ROIs we tested covering virtually the whole brain, the ANCOVA comparing the three groups and controlling for age, sex, education and TIV showed significant group differences [*F*(2,108) = 8.84, *P* = 0.0003, Cohen’s *f* = 0.386, constitutes a medium to large effect size] only in the orbital part of the left inferior frontal gyrus (IFG). Pairwise comparisons revealed significant differences between the aphasic group and healthy controls (−2.76%, *t* = 3.35, *P* = 0.0033), aphasic and non-aphasic stroke groups (−2.87%, *t* = 4.04, *P* = 0.0003), but no difference between the healthy control and non-aphasic participants (−0.11%, *t* = 0.15, *P* = 0.9866) (Fig. 3). The aphasic group showed significant atrophy (2.7%, *t* = 4.57, *P* = 0.0001) that was not observed in either healthy control or non-aphasic stroke groups (Fig. 3). Note that while we analyzed the data using a group approach with specific cut-offs based on the Token Test scores at 3 months, repeating the analysis as a correlation between Token Test scores at 3 months and per cent volume change in the left IFG, including participants who scored 15 on the Token score, also results in a significant association between Token Test performance and IFG volume, *ρ* = 0.36, *P*-value = 0.000148.

Since the Token Test requires adequate attention and memory, as the instructions cannot be repeated, we have checked that the inclusion of verbal memory (HVLT-delay), working memory (Cogstate one-back task) and attention (Cogstate identification task) as covariates did not affect the interpretation of the results. The group ANOVA remained significant with the inclusion of each of these three covariates (*P* = 0.001, *P* = 0.0001 and *P* = 0.0007, respectively). Overall, 17% of participants had recurrent stroke (16% within the aphasic group), removing participants with recurrent stroke from the analysis did not change the results in the left IFG.

Only one participant presented with a lesion in the left orbital IFG (lesion overlapping with the significant ROI) (Supplementary Fig. 1). This participant showed a 12.55% volume decrease in the left orbital IFG over 9 months, likely due to focal neurodegeneration. All reported results remained significant when this participant’s data were removed from the analyses.

Finally, we divided the aphasic patients into two subgroups—those who remained aphasic (≤14 Token score) at 12 months and those who improved their score to >14 (excluding the participant with the lesion in the left IFG), *N* = 12 each. We compared their volume change in the left IFG using a two-tailed two-sample *t*-test (we did not include additional covariates due to a small sample size), and observed a significant difference in brain atrophy, showing 2.7% and 1.2% volume decrease in the not-recovered and recovered groups, respectively, *t*_(2,21)_ = 2.13, *P* = 0.045.

### WM results 3–12 months post-stroke

**Figure 4:**
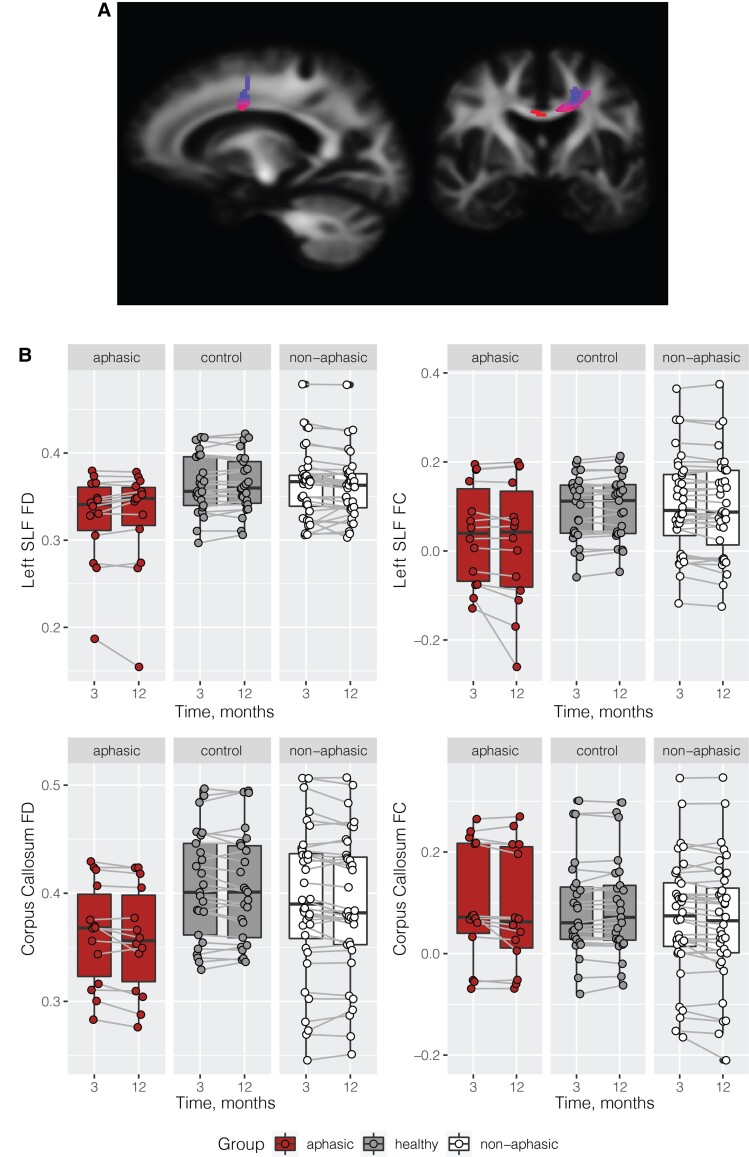
**WM imaging results.** Significant decrease in fibre cross-section in the aphasic versus healthy control group in the corpus collosum and left superior longitudinal fasciculus.

Whole-brain fixel-based analysis in a subset of participants with available diffusion data revealed a significant WM fibre cross-section decrease from 3 to 12 months post-stroke in aphasic participants compared with the healthy control group (Fig. 4A). These group differences were localized in the corpus collosum and the left superior longitudinal fasciculus. No significant group differences were observed in fibre density between aphasic and healthy control groups. No significant differences were observed between the aphasic and the non-aphasic groups, although a similar trend was observed in the left superior longitudinal fasciculus and the corpus callosum, see Fig. 4B. Note that the results between the whole-brain analysis and the tract of interest plots are not identical, namely, we could not extract FC and FD from the exact mask of significant voxels; and the plots do not take covariates [age, TIV and education] into account, but they do demonstrate that the aphasic group has generally lower level of FC and FD in these two tracts and a further decline in FC in the left SLF from 3 to 12 months compared with the healthy control group and the non-aphasic group.

## Discussion

We compared regional brain volume changes from 3 to 12 months in aphasic stroke, non-aphasic stroke and healthy control participants across the whole brain. We observed significant brain volume reduction in the aphasic group in the left IFG, part of the canonical left perisylvian language network. No other brain regions showed significant changes. In addition, we observed a left-lateralized decrease in WM fibre cross-section in aphasic compared with healthy control participants (a subset with available diffusion data), consistent with the location of the reported GM atrophy. These findings obtained with two distinct whole-brain analyses demonstrate GM and WM atrophy in the brain regions associated with a specific behavioural impairment after stroke. Furthermore, they imply that previously reported functional reorganization associated with recovery (e.g. Saur *et al.*^11^) takes place alongside continued atrophy.

Despite functional recovery and behavioural improvement after stroke, the brain continues to shrink.^32^ There is a wealth of evidence for brain volume loss after stroke but the underlying mechanisms that determine the pattern of brain atrophy remain poorly understood. Authors of prior studies have reported brain atrophy due to the expansion of lesions^32^; neurodegenerative processes targeting brain structures known to become vulnerable after stroke, such as the thalamus,^33–35^ or the hippocampus^36,37^ or GM loss predicted by the structural (WM) pathway disruption by lesions.^38^ We demonstrate remote atrophy along the *functional* brain regions subtending language ability in both GM and WM. Methods like lesion-network mapping^39^ suggest that lesions overlapping functional networks could disrupt normal functioning, for example, in depression.^40^ We further show that disrupted functional brain regions might also undergo a structural decline, in this case in a node of the functional language network. We report an average of 2.3% brain volume loss in the area (excluding the participant with a lesion in the orbital IFG showing an extremely fast rate of change), compared with negligible 0.1 and 0.2% change in the non-aphasic and healthy control groups, as well as established expected regular annual rate of average brain volume loss of about 0.2–0.5% in healthy ageing^41–43^ or of 0.95% per year in the decline of ipsi-lesional hemisphere volume after stroke.^32^ This dramatic decline in the orbital IFG was accompanied by an overall improvement in the scores. The differences between the groups at 12 months persisted, suggesting that, compared with healthy controls and non-aphasic stroke patients, on average, participants in the aphasic group were still impaired. However, about half of the participants were no longer aphasic according to the ‘cut-off’ score we used to classify subjects into aphasic or non-aphasic. Those who recovered showed significantly lower volume loss (1.2% compared with 2.7%); however, it was still greater, compared with participants who did not present with aphasic symptoms at 3 months.

The lesions in aphasic and non-aphasic groups had a wide bilateral distribution (Fig. 1), only one subject had an overlapping lesion with the IFG pars orbitalis (Supplementary Fig. 1). This suggests that the observed result was an example of remote atrophy, since lesions in the current study were not confined to the areas typically associated with the left-lateralized perisylvian language network. We further reported a reduction in WM fibre cross-section in the same left frontal brain region (left super longitudinal fasciculus and adjacent corpus collosum). Note that this analysis revealed a spatially localized area of fibre degeneration, which is not likely to reflect general WM neurodegeneration observed in stroke, which tends to be bilateral and extend to both frontal and parietal areas at least at 3 months post-stroke.^25^ Changes in fibre cross-section rather than fibre density were observed, suggesting that the fibre bundle decreased in diameter, possibly following a reduction in fibre density.^44^

In this study, we had access to only a few language tests within the testing battery, but we could ascertain that our aphasic group was impaired on Boston Naming task, as well as semantic and lexical COWAT fluency tests. Our non-aphasic group also showed differences from the healthy controls in lexical fluency. This is consistent with the results of a study showing that a semantic fluency test was sensitive even to mild aphasia, while the lexical fluency task was not correlated with language measures and represents a more general executive functioning test. This is because phonemic or lexical fluency relies more on executive processes and less on the integrity of language networks.^45^ Even then, aphasic patients were significantly more impaired on phonemic fluency compared with non-aphasic participants.

We have interpreted our findings as a disruption to the language network, yet we only observed brain volume loss in the left orbital IFG. Historically, aphasia has been characterized by the disruption to the left inferior frontal (Broca) and superior temporal (Wernicke) areas and roughly mapped to the deficits in production and comprehension. It is now accepted that the picture is more nuanced, and the brain regions involved need to be defined more precisely.^46^ The left IFG pars orbitalis is an integral part of a highly distributed neural network underpinning semantic cognition.^47^ It prominently features as an area observed in semantic studies. In an activation likelihood estimation (ALE) review, it appears in ‘all activation’ studies ALE of 1145 foci and in the ‘general semantic contrasts’ ALE of 691 foci.^48^ Pars orbitalis could be specifically important for the ventral stream semantic processing, linking sound to meaning,^49^ as it is connected to the temporal pole through the uncinate fasciculus.^50^ Controlled semantic processing and working memory are also known to share neural system resources that primarily involve pars orbitalis.^51^ Post-stroke aphasia is characterized not by the amodal semantic deficit as seen in semantic dementia, but by semantic control problems.^52^ Hence, the primary area of vulnerability appears to be the inferior frontal rather than temporal areas. We, therefore, hypothesize that semantic deficits in our cohort and the related volume loss in the orbital part of the left IFG are related to disrupted semantic control in the brain network underlying this function. However, we also acknowledge that the left IFG may be involved in other functions, such as creativity of ideas,^53^ and that previous studies in post-stroke aphasia found changes in brain structure and function outside the canonical language network.^54^

### Limitations

In this study, we did not attempt to describe the neural correlates of specific types of aphasia after stroke, as we lacked a comprehensive assessment of language function in the cohort. Rather, our goal was to determine whether language impairment early after stroke (3 months) is associated with atrophy in the brain regions associated with the language function that is not observed in stroke participants not presenting with specifically language deficits. Future studies should investigate whether different types of aphasia are associated with more specific patterns of structural decline. Furthermore, participants in our study only had relatively small lesions and mild stroke severity, including in the aphasic group, which only presented with a relatively minor language impairment. As the CANVAS study was not specifically focused on aphasia, participants did not require/receive any specific language therapy/rehabilitation over the study period. Future studies should investigate whether a similar pattern of atrophy would be observed in severe aphasia.

Only a much-reduced sample was available for the WM analysis in this study. Yet, a significant result was observed at the whole-brain level, consistent in location with the GM findings. This provides initial multi-modal confirmation of the link between white and GM structural integrity, warranting further investigation of WM structural changes associated with functional reorganization after stroke.

While we reported on specific brain regions showing decline, we cannot determine the mechanism that caused the decline. One approach would be to look at whether in the participants who showed significant atrophy in the orbital part of the left IFG, their lesion was structurally or functionally connected to this ROI, as opposed to participants who did not show significant atrophy in this ROI. Furthermore, in the current study, we used anatomically defined ROIs based on FreeSurfer parcellation. However, future studies could use functionally defined ROIs to specifically elucidate the language network (while participants perform various language tasks) and look at volume changes within this functionally defined network. Finally, an important extension of this work would be following up on the changes beyond 1 year after stroke, to fully understand the trajectory of decline.

## Conclusion

We conclude that language deficits are associated with accelerated structural decline in the functional language network. These findings highlight the complexity of the recovery process by demonstrating that functional reorganization includes not only changes that are associated with functional improvement but also atrophy.

## Supplementary Material

fcac061_Supplementary_Data

## Abbreviations

CANVAS =: Cognition And Neocortical Volume After Stroke
IFG =: inferior frontal gyrus
ROI =: region of interest
SLF =: superior longitudinal fasciculus
