# Supplementary material for: Grey and white matter atrophy 1 year after stroke aphasia
Source: Brain Commun. 2022 Mar 17;4(2):fcac061. doi: 10.1093/braincomms/fcac061 (PMC8971893; doi:10.1093/braincomms/fcac061)
Supplement: fcac061_Supplementary_Data [file fcac061_supplementary_data.zip › Supplementary material.pdf]

**Supplementary material**

**Supplementary Figure 1.** Participant with the lesion (blue) overlapping the IFG pars orbitalis ROI (red).

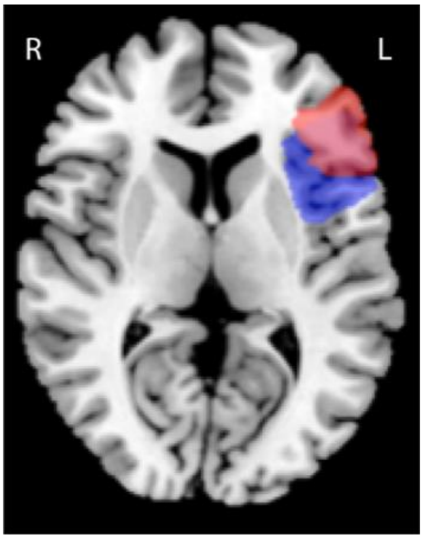

**Supplementary Table 1.** List of ROIs used for analysis.

1. Left bankssts
2. Left caudalanteriorcingulate
3. Left caudalmiddlefrontal
4. Left cuneus
5. Left entorhinal
6. Left fusiform
7. Left inferiorparietal
8. Left inferiortemporal
9. Left isthmuscingulate
10. Left lateraloccipital
11. Left lateralorbitofrontal
12. Left lingual
13. Left medialorbitofrontal
14. Left middletemporal
15. Left parahippocampal
16. Left paracentral
17. Left parsopercularis
18. Left parsorbitalis
19. Left parstriangularis
20. Left pericalcarine
21. Left postcentral
22. Left posteriorcingulate
23. Left precentral
24. Left precuneus
25. Left rostralanteriorcingulate
26. Left rostralmiddlefrontal
27. Left superiorfrontal
28. Left superiorparietal
29. Left superiortemporal
30. Left supramarginal
31. Left frontalpole
32. Left temporalpole
33. Left transversetemporal
34. Left insula
35. Right bankssts
36. Right caudalanteriorcingulate
37. Right caudalmiddlefrontal
38. Right cuneus
39. Right entorhinal
40. Right fusiform
41. Right inferiorparietal
42. Right inferiortemporal
43. Right isthmuscingulate
44. Right lateraloccipital
45. Right lateralorbitofrontal
46. Right lingual
47. Right medialorbitofrontal
48. Right middletemporal

49. Right parahippocampal
50. Right paracentral
51. Right parsopercularis
52. Right parsorbitalis
53. Right parstriangularis
54. Right pericalcarine
55. Right postcentral
56. Right posteriorcingulate
57. Right precentral
58. Right precuneus
59. Right rostralanteriorcingulate
60. Right rostralmiddlefrontal
61. Right superiorfrontal
62. Right superiorparietal
63. Right superiortemporal
64. Right supramarginal
65. Right frontalpole
66. Right temporalpole
67. Right transversetemporal
68. Right insula
69. Left Thalamus
70. Left Caudate
71. Left Putamen
72. Left Pallidum
73. Left Hippocampus
74. Left Amygdala
75. Left Accumbens
76. Right Thalamus
77. Right Caudate
78. Right Putamen
79. Right Pallidum
80. Right Hippocampus
81. Right Amygdala
82. Right Accumbens
