# Supplementary figures and images for: Grey and white matter atrophy 1 year after stroke aphasia
Source: Brain Commun. 2022 Mar 17;4(2):fcac061. doi: 10.1093/braincomms/fcac061 (PMC8971893; doi:10.1093/braincomms/fcac061)

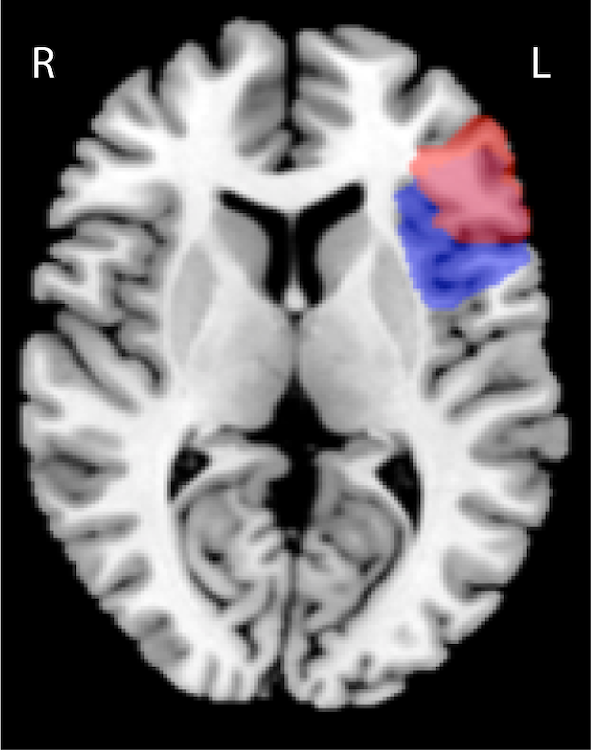

Supplement: fcac061_Supplementary_Data [file fcac061_supplementary_data.zip › Supplementary_Figure 1 full size.tif]
